# Design and Optimization of Pullulan-Isononanoate Films with Bioactive-Loaded Liposomes for Potential Biomedical Use

**DOI:** 10.3390/polym18020305

**Published:** 2026-01-22

**Authors:** Amjed A. Karkad, Aleksandar Marinković, Aleksandra Jovanović, Katarina Simić, Stefan Ivanović, Milena Milošević, Tamara Erceg

**Affiliations:** 1 Faculty of Technology and Metallurgy, University of Belgrade, Karnegijeva 4, 11120 Belgrade, Serbia; amjedkarkad85@gmail.com (A.A.K.); marinko@tmf.bg.ac.rs (A.M.); 2 Faculty of Medical Technology, Elmergib University, Msallatah 7310500, Libya; 3 Institute for the Application of Nuclear Energy INEP, University of Belgrade, Banatska 31b, 11080 Belgrade, Serbia; ajovanovic@inep.co.rs; 4 Institute of Chemistry, Technology and Metallurgy—National Institute of the Republic of Serbia, University of Belgrade, Njegoševa 12, 11000 Belgrade, Serbia; katarina.simic@ihtm.bg.ac.rs (K.S.); stefan.ivanovic@ihtm.bg.ac.rs (S.I.); milena.milosevic@ihtm.bg.ac.rs (M.M.); 5 Faculty of Technology Novi Sad, University of Novi Sad, Bulevar cara Lazara 1, 21000 Novi Sad, Serbia

**Keywords:** pullulan-isononanoate, Liposomes, biopolymer films, silibinin, esterification, smoke tree

## Abstract

This study reports the synthesis and detailed characterization of pullulan-isononanoate (Pull-Iso), as well as the preparation and characterization of Pull-Iso films incorporating liposomes loaded with silibinin (SB) and smoke tree (*Cotinus coggygria*) extract (STExt), to explore the physicochemical and functional properties of pullulan-based biomaterials for potential biomedical applications. Pullulan was successfully esterified with isononanoic acid chloride, as confirmed by ^1^H and ^13^C NMR (Nuclear Magnetic Resonance) and Fourier Transform Infrared (FTIR) spectroscopy. Modification significantly reduced the glass transition temperature (*Tg*), indicating enhanced chain mobility due to the introduction of bulky side chains. Prepared liposomes, embedding SB and extracted smoke tree compounds, exhibited particle sizes ~2000 nm with moderate polydispersity (~0.340) and zeta potential values around –20 mV, demonstrating lower colloidal stability over 60 days, thereby justifying their encapsulation within films. Optical microscopy revealed uniform liposome dispersion in Pull-Iso film with 0.5 g of liposomes, while higher liposome loading (0.75 g of liposomes) induced aggregation and microstructural irregularities. Mechanical analysis showed a reduction in tensile strength and strain at higher liposome content. The incorporation of liposomes encapsulating STExt and SB significantly enhanced the antioxidant activity of Pull-Iso-based films in a concentration-dependent manner, as demonstrated by DPPH and ABTS radical scavenging assays. These preliminary findings suggest that pullulan esterification and controlled liposome incorporation may enable the development of flexible, bioactive-loaded films, which could represent a promising platform for advanced wound dressing applications, warranting further investigation.

## 1. Introduction

The developing field of biopolymer utilization in wound healing addresses the critical need for effective and sustainable medical solutions by leveraging biopolymer-based materials. Derived from natural sources (plants, animals, and microorganisms), these polymers minimize adverse reactions and eliminate the need for invasive removal, thereby reducing medical waste accumulation at the same time. Beyond basic biocompatibility, biopolymers facilitate enhanced healing through tissue regeneration, accelerated wound closure, and reduced scarring, while also serving as platforms for controlled drug delivery. Traditional wound dressings provide only passive protection, as they often fail to create the optimal moist microenvironment required for effective healing. The lack of antimicrobial activity, biocompatibility, and ability to adapt to changes in exudate levels further limits their application [1]. Additionally, due to their limited functionality, they do not permit the controlled release of therapeutic agents, nor do they effectively stimulate tissue regeneration. They also possess reduced mechanical flexibility and lower breathability, which negatively affects patient comfort and the wound healing process [1,2]. Consequently, there has been a surge in research focusing on creating sophisticated biomaterial-based wound dressings to overcome these shortcomings [2]. Ideal wound dressing systems should, in addition to biocompatibility, provide controlled delivery of active compounds in a form that can be transported through the skin barrier. Improved biopolymer films meet these requirements with incorporated active compounds-loaded liposomes. Liposomes are increasingly recognized as a powerful method for delivering active ingredients in sophisticated wound dressings. Their distinctive bilayer structure, formed from phospholipids, enables them to carry a broad spectrum of therapeutic molecules, facilitating their transport through the skin due to the similarity in structure with the cell membrane. These structures have been imposed as a delivery strategy for improving the bioavailability of bioactive compounds [3,4]. With their phospholipid bilayer structure, liposomes can effectively encapsulate both hydrophilic (polar) compounds in their aqueous core and lipophilic (non-polar) compounds within their lipid bilayer [5]. This is crucial for maximizing the therapeutic potential of a broad spectrum of active compounds, such as hydrophobic silibinin (SB) and hydrophilic smoke tree (*Cotinus coggygria*) extract (STExt). SB is well-documented for its potent antioxidant, anti-inflammatory, hepatoprotective, and anti-fibrotic effects. In wound healing, its anti-inflammatory and antioxidant properties can mitigate tissue damage, while its potential anti-fibrotic action could help prevent excessive scarring [6]. However, its poor stability and solubility are significant limitations for its therapeutic use; therefore, incorporating it into delivery systems, such as liposomes and biopolymer matrices, is a crucial prerequisite for implementing it in wound healing [7]. STExt has antioxidant, anti-inflammatory, antigenotoxic, protective, anticancer, and antimicrobial properties [8]. These are crucial for wound healing, as they help combat oxidative stress, reduce inflammation, prevent infection, and promote tissue regeneration [8]. Encapsulation of phenolic bioactives into liposomes before incorporation into a bio-based film enhances molecular protection, improves the skin-active interface by increasing the effective contact surface, and enables sustained and controlled release, thereby mitigating degradation and ensuring prolonged therapeutic exposure [9,10]. Moreover, liposomal loading promotes superior dermal penetration and bioavailability compared to free compounds, as phospholipid vesicles facilitate membrane interaction and diffusion, supporting improved delivery performance once released from the polymer matrix [9,11].

Developing biopolymer-based structures with incorporated bioactive compounds, such as liposomes encapsulating biologically active molecules, represents a significant challenge in wound healing management. This challenge involves ensuring the structural stability of liposomes during their incorporation into the biopolymer matrix and achieving controlled and sustained release of bioactives from the liposomes. Additionally, optimization of the biopolymer–liposome ratio and their mutual interactions is essential to prevent aggregation. Such a system enables a larger effective contact area with the skin, ensures efficient application of liposome-encapsulated bioactive components, and provides adequate mechanical flexibility of the wound dressing system. As a biocompatible and film-forming biopolymer, pullulan has emerged as a promising material for wound dressings. However, despite its favorable properties, its broader application in medicine and pharmaceuticals is limited by its relatively low mechanical strength and high cost. To overcome these limitations, pullulan is frequently blended with other polymers or subjected to chemical modifications to improve its functional performance [12]. Different authors have reported modifications of pullulan using different approaches. Oxidation of pullulan has been applied in order to improve the mechanical properties of this biopolymer and facilitate its combination with other polymers in the design of materials with different purposes [13]. Duceac et al. (2021) [14] have obtained 6-carboxypullulan by oxidation using (2,2,6,6-tetramethyl piperidinyloxy) (TEMPO). Bruneel et al. (1993) [15] first reported periodate oxidation of pullulan. Aldehyde groups incorporated in the pullulan structure in this way enable its combination with polymers and biopolymers with amine groups (chitosan and proteins), forming beads via Schiff bases [16]. Emam et al. (2021) [16] have performed carboxymethylation of pullulan in an aqueous-organic medium at alkaline pH using monochloroacetic acid with the aim of preparing hydrogels in combination with pectin using glutaraldehyde as a crosslinking agent for controllable release of povidone-iodine in wound dressing systems [17]. Enomoto-Rogers et al. (2015) [18] have investigated the influence of acyl chain (C2-C12) on the thermal properties of fully substituted pullulan esters, as well as on the mechanical and morphological properties of films obtained from the pullulan esters. Esterified pullulan for food packaging has been created through two primary methods: pullulan was esterified with various carboxylic acids and octenyl succinic anhydride, and combined with chitosan in the design of food packaging. Carvalho et al. (2020) [19] have reported the chemical modification of the polysaccharide pullulan by grafting poly(3-hydroxybutyrate-co-3-hydroxyvalerate) (PHBHV) and poly(ε-caprolactone) onto its backbone using click chemistry to obtain bio-based materials with potential application as drug delivery systems. As a highly hydrophilic biopolymer, pullulan also presents challenges for certain delivery applications due to its inherent solubility. To address these limitations, we report a novel modification strategy involving the direct esterification of isononanoyl chloride onto the pullulan backbone, yielding a branched biopolymer. This precise and controllable reaction route enables fine-tuning of pullulan solubility, chain mobility, and interfacial behavior, allowing the design of a biopolymer that simultaneously exhibits enhanced hydrophobicity and preserved aqueous processability, an uncommon and technologically valuable combination for biomedical and other applications.

To the best of our knowledge, this study presents the first report on the direct esterification of pullulan with isononanoic acid chloride and its subsequent integration into liposome-loaded bioactive film systems. The work focuses on the synthesis, structural elucidation, and thermal characterization of a newly developed pullulan-isononanoate (Pull-Iso), providing fundamental insight into how branched hydrophobic modification governs the physicochemical behavior of pullulan. The modification strategy was designed to operate at the solubility threshold, achieving a degree of esterification sufficient to enhance hydrophobicity while preserving aqueous processability, thereby enabling film formation and bioactive loading. Building on this foundation, flexible Pull-Iso films were developed and systematically characterized following the incorporation of liposomes encapsulating bioactive compounds of distinct polarities, namely SB and STExt. The overarching aim is to establish a multifunctional and stable biopolymer platform capable of synergistically delivering hydrophilic and hydrophobic bioactives through optimized liposome–biopolymer integration. Liposomal encapsulation is expected to improve stabilization of polyphenolic constituents and to promote sustained release and dermal permeation, thereby maximizing antioxidant, anti-inflammatory, and dermato-protective activity. Embedding these liposome-loaded systems into bio-based polymer films further provides a biodegradable platform for controlled topical delivery. Beyond the novelty of the chemical modification itself, this study introduces a previously unreported material concept, namely the incorporation of liposomes loaded with active compounds into chemically modified pullulan-based films. To our knowledge, this is the first demonstration of liposome-encapsulated bioactives integrated within a hydrophobically modified pullulan matrix, resulting in a multifunctional polymer–liposome hybrid platform that combines controlled bioactive delivery, tailored surface properties, and mechanical flexibility. This dual-level novelty establishes a new class of bio-based composite materials with strong potential for advanced wound-care systems, dermal patches, and functional packaging applications.

## 2. Materials and Methods

### 2.1. Materials

Pullulan (Mw~574,570 g/mol) was procured from Avena Lab (Vršac, Serbia). Isononanoic acid was supplied from SOLECHEM S.R.L. (Milan, Italy), while thionyl chloride, 2-pyrrolidone, triethylamine, and dimethylformamide (DMF) were supplied from Thermo Scientific Chemicals (Waltham, MA, USA). Glycerol and ethanol were supplied from Centrohem (Stara Pazova, Serbia). Potassium hydroxide and hydrochloric acid were from BASF SE (Ludwigshafen, Germany). Phospholipon 90G (phosphatidylcholine from soybean) was from Nattermann Phospholipids (Cologne-Bocklemünd, Germany). Silibinin (≥98%, HPLC grade) was supplied by Sigma Aldrich (Steinheim, Germany). Highly comminuted plant material (the wooden part of *C. coggygria*, collected in Belgrade, Serbia) was used for the extract preparation. ABTS (2,2′-azino-bis (3-ethylbenzothiazoline-6-sulphonic acid)), vitamin C (ascorbic acid), and DPPH (2,2-diphenyl-1-picrylhydrazyl) were purchased from Sigma Aldrich (Steinheim, Germany), while potassium persulfate and dimethyl sulfoxide (DMSO) were purchased from Sigma-Aldrich (Darmstadt, Germany).

### 2.2. Modification of Pullulan

To obtained esterified pullulan at its solubility threshold—achieving a degree of esterification sufficient to enhance hydrophobicity while retaining water solubility—the following procedure was applied. Isononanoic acid chloride (3,5,5-trimethylhexanoyl chloride) was synthesized by reacting isononanoic acid (3,5,5-trimethylhexanoic acid) with thionyl chloride in a 1:3 molar ratio. The reaction temperature was gradually increased from ambient temperature to 70 °C and maintained at this temperature for 8 h. After completion of the reaction, the excess thionyl chloride was removed by distillation, followed by vacuum distillation of the product at 106–108 °C under 20 mmHg. This procedure yielded isononanoic acid chloride with a purity of 95%.

Pullulan (Pull) (19.55 g) was dissolved in 200 mL of a 3:1 (*v*/*v*) mixture of 2-pyrrolidone and DMF at room temperature. Triethylamine (15.19 mL, 1.0 equivalent) was added to the solution. The reaction flask was cooled using an ice bath, and isononanoyl chloride (19.17 g, 1.0 equivalent) was then added dropwise over 2 h. The reaction mixture was continuously stirred at 25 °C for 12 h, and finally at 40 °C for 2 h. After completion of the reaction, the reaction mixture was transferred into a 2 L beaker equipped with a mechanical stirrer and a Teflon propeller-type mixer (IKA, Staufen, Germany). At high stirring (~1000 rpm), 1 L of methanol was added dropwise for 2 h until a white fibrous-like product was separated. The product was purified three times in the following manner: after dissolution in 200 mL of water under mixing at 30 °C for 3 h, it was transferred into a beaker with a mechanical stirrer and subsequently precipitated by the addition of 1 L of methanol for 2 h at 1000 rpm. Finally, the product named Pull-Iso was dried in an oven at 50 °C for 24 h, and under vacuum at 50 °C/2000 Pa for 16 h.

### 2.3. Preparation of Smoke Tree Extract

The extract from the smoke tree wood (STExt) was prepared by subjecting 3 g of finely ground plant material to ultrasound-assisted extraction in 120 mL of 80% (*v*/*v*) ethanol [20]. The extraction was performed in a Sonorex Super RK ultrasonic bath (Bandelin, Berlin, Germany) for 30 min. Following extraction, the mixture was filtered through fine-grade filter paper, and the resulting filtrate was used for subsequent liposome formulation. The extraction yield amounted to 12%.

### 2.4. Preparation of Liposomes with Smoke Tree Extract and Silibinin and Determination of the Encapsulation Efficiency

Multilamellar liposome vesicles containing STExt and SB were prepared using the proliposome method, following a previously described procedure [21]. Briefly, 6 g of phospholipids (Ph) were mixed with 60 mL of ethanol-based STExt and 1.5 g of SB. The mixture was stirred and heated between 50 and 60 °C for 15 min. After cooling to room temperature (25 °C), 120 mL of ultrapure water was slowly added. The dispersion was stirred for another hour at 800 rpm. Complete evaporation of ethanol during the process resulted in a final phospholipid dispersion concentration of 50 mg/mL.

The encapsulation efficiency (EE) of SB and STExt polyphenols into liposomes was evaluated using an indirect method, based on the quantification of non-encapsulated compounds in the supernatant. EE (%) was calculated according to Equation (1):

(1) $$EE\;\left(\%\right)=\;\frac{TPCi-TPCsup}{TPCi\;}\times 100$$

The TPC_i_ represents the total polyphenol content initially present in the STExt, as well as the total content of SB, used for liposome preparation, and TPC_sup_ corresponds to the total polyphenols (STExt polyphenols and SB) measured in the supernatant.

Non-encapsulated polyphenols were separated from the liposomal suspension by centrifugation at 17,500 rpm and 4 °C for 45 min using a Thermo Scientific Sorval WX Ultra series ultracentrifuge (Thermo Fisher Scientific, Waltham, MA, USA). The total polyphenol content in both the initial extract and the collected supernatants was determined via the modified Folin–Ciocalteu assay, with absorbance readings taken at 765 nm [22].

### 2.5. Characterization of the Liposomal Dispersion—Particle Size, Size Distribution, and Zeta Potential

The average particle size, polydispersity index (PDI), and zeta potential of the liposomal formulation were determined using photon correlation spectroscopy (PCS). Measurements were conducted using a Zetasizer Nano ZS (Nano Series, Malvern Instruments Ltd., Malvern, UK), capable of analyzing particles in the range of 0.6 nm to 6 µm. All analyses were performed at 25 °C after a 500-fold dilution of each sample with ultrapure water. Each sample was measured in triplicate, and results are expressed as mean values. For stability assessment, the aforementioned physicochemical parameters of SB- and STExt-loaded liposomes were monitored over a 60-day storage period at 4 °C. Measurements were repeated on the 1st, 7th, 14th, 30th, and 60th day using the same PCS method. Measurements were conducted in triplicate, and statistical evaluation was performed using STATISTICA software, version 7.0. One-way analysis of variance (ANOVA) was applied to assess differences, followed by Duncan’s multiple range test. Results are expressed as mean ± standard deviation. Statistical significance was established at a confidence level of *p* < 0.05.

### 2.6. Nuclear Magnetic Resonance (NMR) Spectroscopy

Nuclear magnetic resonance spectroscopy (NMR)—^1^H and ^13^C NMR spectra were recorded on an Agilent/Varian 400-MR NMR spectrometer (399.74 MHz for ^1^H and 100.53 MHz for ^13^C) equipped with a 400-MR console and a ^1^H/^19^F/X5 mm PFG ATB broadband probe. The spectra were acquired at 298 K in DMSO-d6. Chemical shifts (δ) are reported relative to the residual solvent signal (δH = 2.50 ppm and δC = 39.52 ppm). Spectral processing and analysis were performed using MestReNova software, version 14.2.0.

### 2.7. Determining the Degree of Esterification (EV) by Potentiometric Titration

To determine the degree of esterification of pullulan by the potentiometric titration method, the content of free carboxylic groups was first measured, and then, after the hydrolysis of all esterified groups, the total content of carboxylic acids was determined. Acid value (AV) was determined according to the standard method ASTM D3644 [23]. A potentiometric titration of Pull-Iso, dissolved in a mixture of water and DMSO, was performed with 0.1 N KOH. AV was determined using the following Equation (2):

(2) $$AV=\frac{56.1\times c_{\mathrm{KOH}}\times V_{\mathrm{KOH}}}{m_{\mathrm{Pull-Iso}}}$$

where $c_{\mathrm{KOH}}$ is the concentration of KOH (0.1 mol/L KOH), V is the volume of KOH standard solution, and $m_{\mathrm{Pull-Iso}}$ is the mass of Pull-Iso.

After the determination of free acids, the sample was subjected to saponification. Determination of saponification value (SV) was performed according to ISO 3657:2023 [24]. The principle of determination is based on basic hydrolysis and neutralization of all ester and acidic groups, respectively, using KOH solution. The titration was performed with HCl standard solution (0.1 mol/L). SV was calculated according to Equation (3):

(3) $$SV=\frac{56.1\times c_{\mathrm{HCl}}\times \left(V_{\mathrm{HCl}0}-V_{\mathrm{HCl}}\right)}{m_{\mathrm{Pull-Iso}}}$$

where *V*_HCl0_ is a volume of HCl used for neutralization of KOH solution (blank titration), and *V*_HCl_ is a volume of HCl used for neutralization of Pull-Iso solution after treatment with KOH.

### 2.8. Differential Scanning Calorimetry (DSC) Analysis

Thermal properties of Pull-Iso were analyzed using differential scanning calorimetry (DSC) on a TA Instruments Q20 device (New Castle, DE, USA) under a nitrogen atmosphere with a flow rate of 50 mL/min. The instrument was calibrated for temperature and cell constant using an indium standard. The sample was initially heated from room temperature to 250 °C at a rate of 10 °C/min. The glass transition temperature (*Tg*) was identified as the midpoint of the heat capacity change (*∆Cp*). The standard uncertainty for temperature measurements was *u*(*T*) = 0.5 °C. Data processing and presentation were conducted using the TA Analyzer software, version 11.0.

### 2.9. Preparation of Films

Neat films were prepared by dissolving 2 g of Pull-Iso in 25 mL of distilled water. To this solution, 0.2 g of glycerol was added and stirred until complete dissolution. The resulting Pull-Iso solution was then cast into a Petri dish and dried at 50 °C until a constant weight was achieved, indicating complete solvent evaporation. Films loaded with liposomes (Pull-Iso-Lip) were prepared identically, with the key difference being the addition of 0.25 g, 0.5 g, and 0.75 g (in the text assigned as Pull-Iso-Lip 1, 2, and 3, respectively) liposome dispersion with encapsulated SB and STExt to the biopolymer solution, followed by homogenization at room temperature. Freshly prepared liposomes with an average diameter of approximately 1979 nm were used for film preparation and were incorporated into the polymer matrix on the first day after preparation. The larger size values (>4000 nm) reported during storage correspond exclusively to the stability study and were not applied in the final wound-dressing formulation. Due to the high overall EE of polyphenols, the entire liposomal suspension, without prior separation of a non-encapsulated fraction, was used for film preparation.

### 2.10. Fourier Transform Infrared Spectroscopy (FTIR) Analysis

Fourier transform infrared (FTIR) spectra were recorded with a Nicolet™ iS™ 10 FT-IR Spectrometer (Thermo Fisher SCIENTIFIC) with Smart iTR™ Attenuated Total Reflectance (ATR) Sampling accessories (Waltham, MA, USA). The spectra were recorded in the range 4000–500 cm^−1^, in 20 scans mode, and at a resolution of 4 cm^−1^.

### 2.11. Optical Microscopy

Liposome-loaded films were examined using a Delta Optic Smart 5.0 MP digital optical microscope (Delta Optical, Novi Osinač, Poland), and images were captured with HiView software (HIRISE) version 2.3.

### 2.12. Water Contact Angle (WCA) Measurements

The surface wettability of the films was evaluated by the sessile drop method. Distilled water droplets were deposited onto the film surfaces at ambient temperature (23 ± 2 °C). The contact angles were determined using Ossila Contact Angle software v1.3.0.0 (Sheffield, UK), and the mean values were calculated from five independent measurements for each sample to ensure reliability.

### 2.13. Moisture Content (MC), Total Soluble Matter (TSM) Analysis, and Swelling Test

The stability of the prepared films was evaluated through measurements of moisture content (MC), total soluble matter (TSM), and swelling ratio (SR). The MC was determined by drying film samples (10 × 10 mm) in an oven at 105 ± 2 °C until a constant weight was achieved. The reduction in mass during drying was attributed to the evaporation of water from the films.

The TSM was quantified as the percentage of the film’s dry mass that dissolved in 25 mL of buffer solution over 24 h (modified procedure described by Erceg et al., 2025 [25]). Three specimens of each film (10 × 10 mm) were initially weighed (m_1_) and then immersed in the buffer pH 5.5 (simulating the pH of the skin) at room temperature for 24 h. After incubation, the remaining undissolved portions were filtered, dried in an oven at 105 ± 2 °C until reaching a constant weight, and reweighed (m_2_). The TSM value for each sample was calculated using Equation (4):

(4) $$TSM=\;\frac{m_{1}-m_{2}}{m_{1}}\;\times 100\%$$

The swelling degree (SD) was evaluated by immersing pre-weighed film samples (10 × 10 mm) in 25 mL of buffer pH 5.5 at room temperature for 30 min. After the immersion period, the swollen films were carefully removed, surface moisture was gently blotted off, and the samples were weighed again (modified procedure described by Apriliyani et al., 2020 [26]. All measurements were performed in triplicate, and the swelling degree (SD) was calculated according to Equation (5):

(5) $$SD=\;\frac{m_{2}-m_{1}\;}{m_{1}}\times 100\%$$

where m_2_ is the weight of the sample in the swollen state (after 30 min), and m_1_ is the weight of the dry sample.

### 2.14. Analysis of Mechanical Properties

The mechanical properties of both neat film and films incorporating liposomes were analyzed using an Autograph AG-X Plus High-Speed Universal Testing Machine (Shimadzu, Kyoto, Japan) at room temperature. Rectangular samples, measuring 50 × 10 mm with a thickness of up to 1 mm, were tested at a speed of 1 mm/min. All measurements were performed in triplicate to ensure reliability.

### 2.15. Determination of Antioxidant Potential of Developed Films

The antioxidant capacity of liposomes with SB and STExt and prepared films was evaluated using two complementary chemical assays, namely the ABTS and DPPH radical scavenging tests.

#### 2.15.1. ABTS Assay

The ABTS radical scavenging activity of the developed neat film and films with liposomes containing bioactive compounds (SB and STExt) was determined according to the method described by Zuhair et al. [27] with minor modifications. The ABTS^•+^ radical cation was generated by mixing an ABTS solution (0.019 g of ABTS dissolved in 5 mL of distilled water) with potassium persulfate solution (88 µL), followed by incubation for 24 h at 4 °C in the dark. The resulting ABTS^•+^ working solution was diluted with distilled water to obtain an absorbance of approximately 0.700 ± 0.020 at 734 nm (UV Spectrophotometer UV-1800, Shimadzu, Kyoto, Japan). For the assay, 2.8 mL of the ABTS^•+^ solution was mixed with 200 µL of the dissolved film (30 mg of film in 2 mL of distilled water) or 200 µL of the liposome system diluted with water in a ratio of 1:10. After 6 min of incubation at room temperature, the absorbance was measured at 734 nm. The radical scavenging activity was calculated using Equation (6):

% inhibition = (A_0ABTS_ − A_xABTS_) × 100/A_0ABTS_ (6)

where A_0ABTS_ represents the absorbance of the ABTS^•+^ solution alone, and A_xABTS_ corresponds to the absorbance of the ABTS^•+^ solution in the presence of bio-based film. Ascorbic acid was used as a positive control.

#### 2.15.2. DPPH Assay

The antioxidant capacity of the neat Pull-Iso film and bio-based films with SB and STExt-loaded liposomes was also evaluated using the DPPH free radical scavenging assay, based on their hydrogen-donating ability [27]. Briefly, 200 µL of dissolved film (30 mg of film in 2 mL of distilled water) or 200 µL of the liposome system diluted with water in a ratio of 1:10 was mixed with 2.8 mL of an ethanolic DPPH^•^ solution adjusted to an absorbance of approximately 0.800 at 517 nm (7 mg of DPPH powder in 250 mL of ethanol). After incubation for 20 min in the dark at room temperature, the absorbance was measured at 517 nm. The percentage of DPPH^•^ radical inhibition was calculated using Equation (7):

% inhibition = (A_0DPPH_ − A_xDPPH_) × 100/A_0DPPH_ (7)

where A_0DPPH_ is the absorbance of the control solution, and A_xDPPH_ is the absorbance of the DPPH^•^ solution in the presence of the sample. Ascorbic acid served as the positive control.

## 3. Results and Discussion

### 3.1. Results of NMR Spectroscopy Analysis

**Figure 1:**
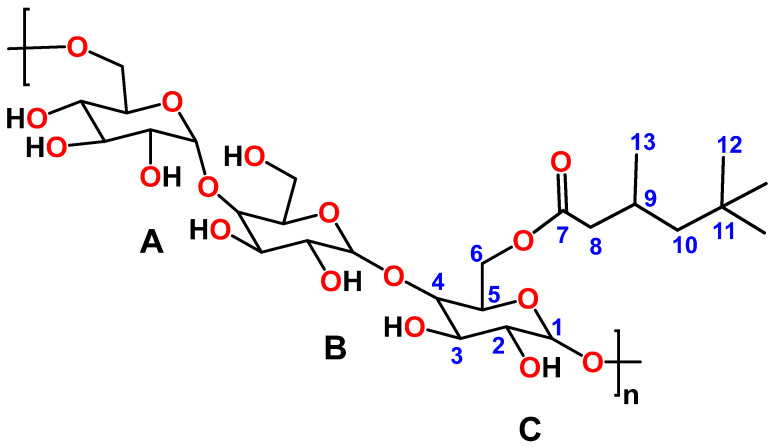
Structure of pullulan-isononanoate (Pull-Iso).

**Figure 2:**
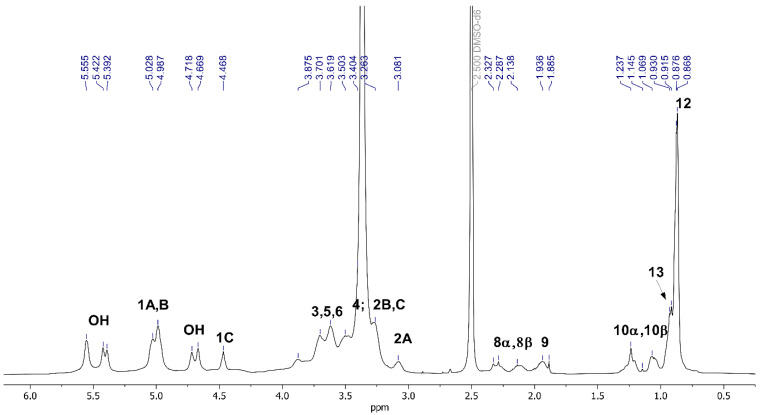
^1^H NMR spectrum of pullulan-isononanoate (Pull-Iso).

**Figure 3:**
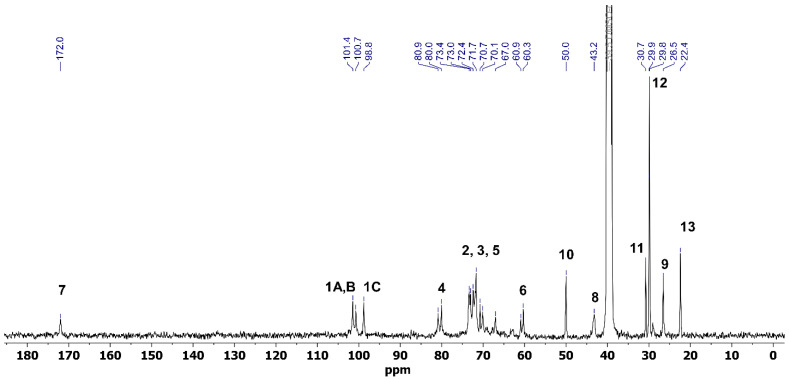
The ^13^C NMR spectrum of pullulan-isononanoate (Pull-Iso).

The ^1^H and ^13^C NMR analyses were employed to confirm the successful synthesis of Pull-Iso, whose structure is presented in Figure 1. NMR spectroscopy has previously been used for pullulan and isononanoic acid structure analysis [28] and has proven to be an indispensable tool for this type of study (Figures S3 and S4, Supplementary Materials). The pullulan and isononanoic acid structures are presented in Figures S1 and S2 are labeled with residues A, B, and C, connected via α-(1→4) D-glucosidic units, which are themselves linked through α-(1→6) D-glucosidic units, and are consistent with the assignment in previous reports [28,29,30]. The modification with isononanoic acid was confirmed by one-dimensional (1D) and two-dimensional (2D) NMR spectroscopy and comparison with literature data [31,32,33]. The ^1^H and ^13^C NMR spectra of the modified biopolymer recorded in DMSO-*d*_6_ are presented in Figure 2 and Figure 3. The 2D experiments are shown in Supplementary Figure S6, where the successful esterification of pullulan with isononanoic acid chloride was confirmed. The signal at *δ*_H_ 0.83 corresponds to the terminal methyl protons (3H, H-12) of the isononanoate moieties, while the signal of the methyl group H-13 (3H) appears at *δ*_H_ 0.92 (Figure 2 and Figure S2). The signals in the *δ*_H_ 1.00–1.30 range are assigned to the methylene protons (H-10α and H-10β) within the aliphatic chain. The signals at *δ*_H_ 2.13 and 2.32 (2H, H-8α and H-8β) correspond to the methylene group in the α-position relative to the carbonyl group, while *δ*_H_ 1.94 corresponds to the H-9 (1H). Broad signals in the *δ*_H_ 3.00–4.00 region are attributed to ring protons, whereas those observed between *δ*_H_ 4.25–5.75 correspond to anomeric and hydroxyl protons of the pullulan backbone, characteristic of α-(1→4) and α-(1→6) glycosidic linkages (Figure 2 and Figure S3). The presence of free hydroxyl protons provides evidence for the partial esterification of the accessible hydroxyl groups. The signals at *δ*_H_ 5.02 and 4.99 in the ^1^H NMR spectrum correspond to CH groups and are assigned to the anomeric proton H-1A and H-1B, respectively, while H-1C is observed at *δ*_H_ 4.47 (Figure 2 and Figure S3).

The ^13^C NMR spectrum of Pull-Iso shows characteristic signals for both the carbohydrate and aliphatic domains. Resonances in the range *δ*_C_ 22.2–50.6 correspond to the carbons of methyl, methylene, and methine groups in the isononanoate chains (Figure 2 and Figure S4). Signals in area *δ*_C_ 60.3–101.4 are assigned to oxygenated carbons of the pullulan backbone, including anomeric carbons, confirming the preservation of the polysaccharide structure (Figure 2 and Figure S3). The signal observed at *δ*_C_ 172.0 is attributed to the carbonyl carbon of ester groups, providing direct evidence of the successful incorporation of isononanoate units into the pullulan matrix (Figure 2 and Figures S2, S4 and S6). Overall, the combination of 1D and 2D NMR spectral data unequivocally confirms the formation of isononanpullulan through esterification of pullulan hydroxyl groups with isononanoic acid.

The Pull-Iso sample exhibited an SV of 264 mg KOH/g, as determined by the procedure described in Section 3.2. The corresponding EV, obtained from the difference between SV and AV, was 252 mg KOH/g, indicating that about 0.81 hydroxyl group per anhydroglucose unit was esterified with isononanoic acid.

### 3.2. Results of FTIR Analysis

**Figure 4:**
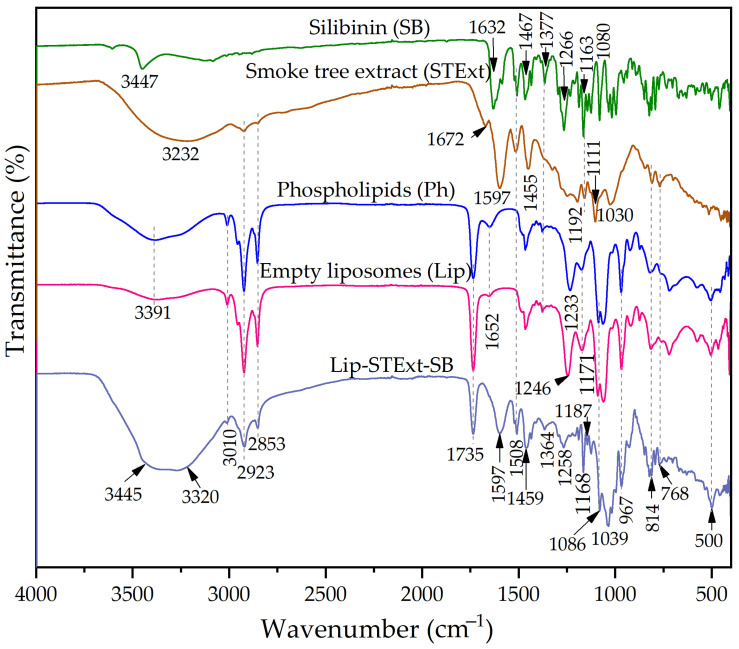
FTIR spectra of silibinin (SB), smoke tree extract (STExt), phospholipids (Ph), empty, and SB and STExt-loaded liposomes.

The spectra of SB, STExt, phospholipids, empty liposomes, and liposomes loaded with STExt and SB are provided in Figure 4. In the FTIR spectrum of SB, a broad band observed in the region of 3400–3200 cm^−1^ corresponds to the O–H stretching vibrations arising from the phenolic and alcoholic hydroxyl groups (the molecular structure of SB is shown in Supporting Information, Figure S5b). A weaker absorption band assigned to aromatic C–H stretching appears at 3050–3000 cm^−1^. The two distinct bands located between 2900 cm^−1^ and 2800 cm^−1^ are characteristic of the asymmetric and symmetric stretching vibrations of aliphatic CH_2_ and CH_3_ groups. A pronounced band at 1632 cm^−1^ is attributed to the C=O stretching vibration of the keto group. The absorption features around 1600 cm^−1^, 1510 cm^−1^, and 1450 cm^−1^ originate from aromatic ring C=C skeletal vibrations, along with CH_2_ bending modes. The region between 1270 cm^−1^ and 1050 cm^−1^ corresponds to C–O stretching vibrations, associated with the ether, phenolic, and alcoholic functionalities present in the molecule. Finally, the bands detected in the 900–700 cm^−1^ region are assigned to out-of-plane deformation vibrations of aromatic C–H bonds. The FTIR spectrum of the STExt exhibits a complex profile, reflecting the presence of diverse phytochemical constituents such as flavonoids, phenolic acids, tannins, and other polyphenolic compounds. A broad absorption band observed in the region of ~3300–3400 cm^−1^ is indicative of O–H stretching vibrations, originating from phenolic and alcoholic hydroxyl groups. The asymmetric and symmetric stretching vibrations of aliphatic –CH_3_ and –CH_2_ groups appear as distinct peaks at ~2920–2950 cm^−1^. The aromatic C=C skeletal stretching vibration associated with flavonoids and other polyphenolic structures is detected as the peak at 1672 cm^−1^. Additional bands at 1597 cm^−1^ and 1508 cm^−1^ correspond to C=C stretching within aromatic rings, which is often linked to the presence of condensed tannins. The C–H bending vibrations of aliphatic –CH_2_ groups and aromatic structures manifest as peaks at 1455 cm^−1^, while the band at ~1370–1380 cm^−1^ is attributed to O–H bending coupled with C–H deformation vibrations. The C–O stretching vibrations of phenolic –OH groups appear in the region of ~1230–1260 cm^−1^. Meanwhile, C–O–C and C–O stretching bands associated with ethers, alcohols, and polysaccharides are evident at 1030 cm^−1^ and 1086 cm^−1^. Finally, characteristic bands at 967 cm^−1^ and 814 cm^−1^ are assigned to aromatic C–H out-of-plane bending vibrations, consistent with substituted benzene ring systems typical of polyphenolic compounds.

Phospholipids are amphiphilic biomolecules composed of a glycerol backbone esterified with two fatty acid chains and a phosphate-containing polar head group (Figure S5a, Supporting Material). The FTIR spectrum of the phospholipid displays characteristic absorption bands associated with its aliphatic and phosphate-containing functional groups. The peak at 3010 cm^−1^ corresponds to =C-H and =CH_2_ stretching. The region of ~2920–2850 cm^−1^ corresponds to the asymmetric and symmetric C–H stretching vibrations of methylene (–CH_2_) groups, which are typical of long fatty acid hydrocarbon chains. A distinct band observed at 1735 cm^−1^ is attributed to the C=O stretching vibration of ester carbonyl groups, confirming the presence of ester linkages in the glycerol backbone. The CH_2_ scissoring (bending) vibrations appear at ~1465–1470 cm^−1^, while the band at ~1370–1380 cm^−1^ is associated with symmetric bending of CH_2_ groups, indicating the terminal methyl groups of fatty acid chains, and O-H bending (in-plane) originating from alcohol and phenolic groups. A pronounced absorption band at 1246 cm^−1^ corresponds to the asymmetric stretching of P=O bonds, characteristic of the phosphate headgroup of phospholipids. The FTIR spectrum of empty liposomes prepared from phospholipids exhibits the same characteristic absorption bands as those observed for the pure phospholipid material. This confirms that the liposome formation process does not induce any detectable chemical modification of the phospholipid structure. The FTIR spectra of SB and STExt-loaded liposomes show noticeable peak shifts and the appearance of additional absorption bands, confirming the successful incorporation of the active compounds into the liposomal structure. The bands at 3345 and 3320 cm^−1^ are assigned to the O–H stretching vibrations of phenolic and alcoholic groups present in SB and phytochemicals from the STExt. The absorption peaks observed at 1597 cm^−1^, 1508 cm^−1^, and 1459 cm^−1^ correspond to the C=C stretching vibrations of aromatic rings, characteristic of tannins and other polyphenolic constituents, as well as C–H bending vibrations of aliphatic –CH_2_ groups associated with aromatic structures. Although some FTIR bands of SB overlap with those of the plant extract, the slight shifts in peak positions indicate specific interactions between the extract compounds and the liposomal surface. These spectral changes suggest that the hydrophilic phenolic constituents preferentially associate with the outer polar region of the lipid bilayer, supporting the hypothesis of surface binding rather than encapsulation within the hydrophobic lipid core.

**Figure 5:**
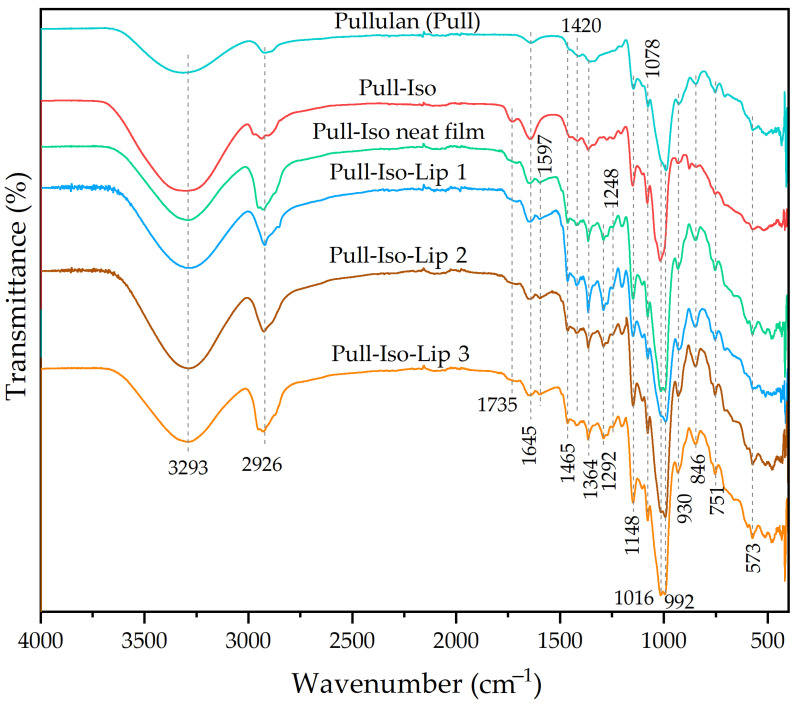
FTIR spectra of pullulan (Pull), pullulan-isononanoate (Pull-Iso), Pull-Iso neat, and liposome-loaded Pull-Iso-based films.

The FTIR spectrum of neat pullulan exhibited a broad absorption band around ~3300 cm^−1^, corresponding to O–H stretching vibrations of hydroxyl groups abundant in the glucose repeating units (Figure 5). The bands observed between 2920 cm^−1^ and 2800 cm^−1^ are assigned to the asymmetric and symmetric C–H stretching vibrations of –CH_2_ groups present in the polysaccharide backbone. The peaks at ~1410–1420 cm^−1^ and ~1370–1380 cm^−1^ were associated with C–H bending (scissoring and deformation) vibrations of methylene groups. The prominent band in the region of ~1155–1160 cm^−1^ corresponded to C–O–C asymmetric stretching, confirming the presence of glycosidic linkages characteristic of α-glucan structures. Additionally, the bands at 1100–1020 cm^−1^ were linked to C–O stretching and C–O–C skeletal vibrations. A signal around ~920–950 cm^−1^ indicated α-1,6 glycosidic bond vibrations, while the peak at ~850–870 cm^−1^ further confirmed α-linked glucose units typical of pullulan. Upon modification to Pull-Iso, a noticeable decrease in the intensity of the –OH stretching band (~3300 cm^−1^) was observed, indicating partial esterification of hydroxyl groups. Simultaneously, new absorption bands emerged in the range of ~1720–1735 cm^−1^, corresponding to C=O stretching vibrations of ester groups, confirming the successful chemical grafting of isononanoate chains. Enhancement of C–H stretching vibrations near ~2920–2850 cm^−1^ further supported the introduction of aliphatic chains from the isononanoate moiety. The shift and slight reduction in the glycosidic C–O–C stretching bands (~1150–1020 cm^−1^) suggested structural rearrangement due to substitution on the pullulan backbone.

The FTIR profiles of films prepared from neat Pull-Iso and from Pull-Iso containing liposomes loaded with SB and STExt retained all characteristic ester-associated peaks, indicating preserved modification after film formation (Figure 5). However, broadened –OH bands and slight shifts in C–O–C stretching regions implied the establishment of intermolecular hydrogen bonding and physical interactions within the film matrix. A characteristic band at 1645–1650 cm^−1^ appearing in spectra of films is attributed to H–O–H bending vibrations of adsorbed moisture.

### 3.3. Results of DSC Analysis of Pull-Iso

**Figure 6:**
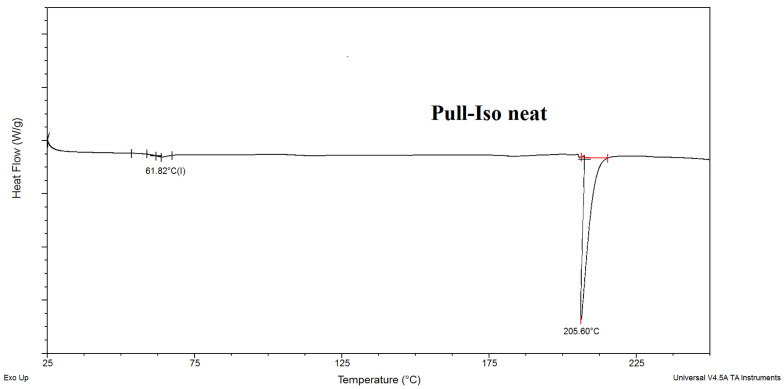
DSC curve for neat pullulan-isononanoate (Pull-Iso).

The DSC thermogram of neat Pull-Iso was recorded to evaluate the influence of esterification on the phase transition behavior (Figure 6). The glass transition temperature (*T_g_*) of Pull-Iso was observed at approximately 61 °C, which is significantly lower than that of neat pullulan (typically above 120 °C, depending on its moisture content and molecular weight) [34]. The reduction in glass transition temperature is attributed to the branching of the pullulan backbone, which introduces free volume within the polymer matrix and consequently increases segmental chain mobility. Pullulan is an amorphous polysaccharide and, due to the absence of crystalline domains, does not exhibit a true melting point [35]. An increase in the acyl carbon number led to a progressive decrease in the glass transition temperature of the pullulan esters, indicating enhanced chain mobility due to the incorporation of longer, more flexible side groups. Esterification of pullulan with isononanoic acid performed in this study yielded a polymer exhibiting a *T_g_* value between that of pullulan valerate (67 °C) and pullulan hexanoate (46 °C), as reported by Enamoto-Rogers et al. (2015) [18], while for pullulan octanoate and decanoate, these values were 42 °C and 35 °C, respectively. This result is consistent with expectations, considering that isononanoic acid contains six carbon atoms in its main chain. Consequently, the thermal properties of pullulan esters can be precisely fine-tuned within a narrow range by modifying the structure of the acyl halide or carboxylic acid used for esterification. A pronounced endothermic peak at around 205 °C in the Pull-Iso thermogram corresponds to the onset of thermal degradation of the esterified polymer.

### 3.4. Results of EE and Particle Size, Size Distribution, and Zeta Potential via Storage Stability Study

The EE of total polyphenols, including SB and polyphenol components from the STExt, was determined using the Folin–Ciocalteu assay. The results confirmed that a substantial fraction of the bioactive compounds is successfully incorporated within the liposomal carriers (87.1 ± 1.4%), indicating that most extract components were incorporated into the liposomal system. Due to the high encapsulation efficiency, the entire liposomal dispersion containing the total amount of added extract and SB was incorporated into the films without separation of any non-encapsulated fraction. Although release kinetics were not assessed in the present study, the high EE indicates effective retention of bioactives within the vesicles.

**Figure 7:**
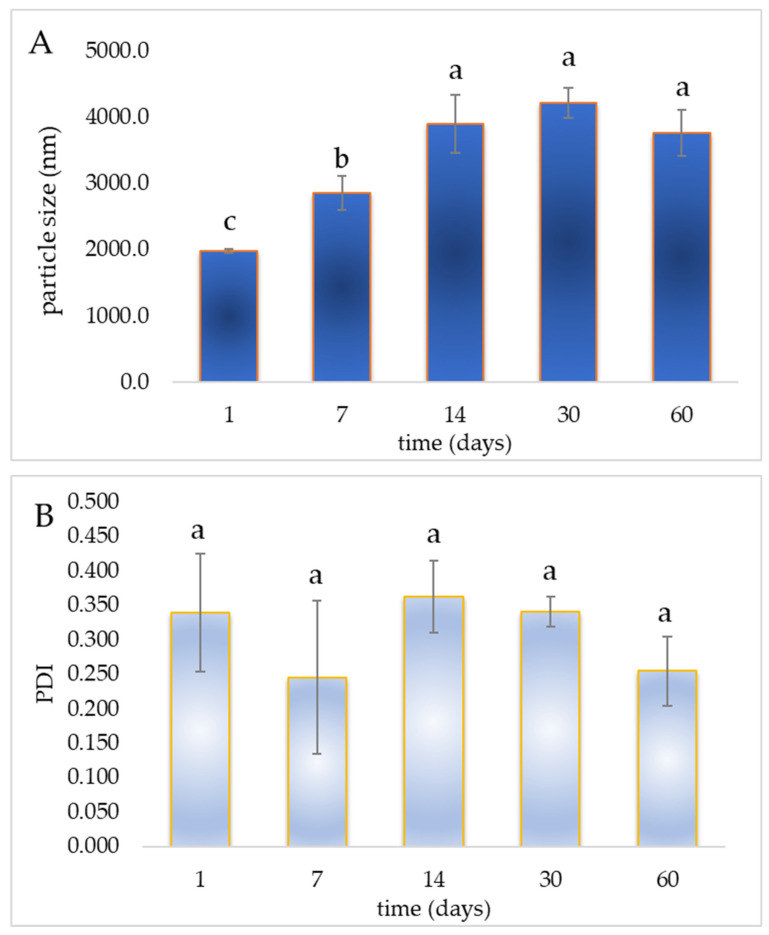

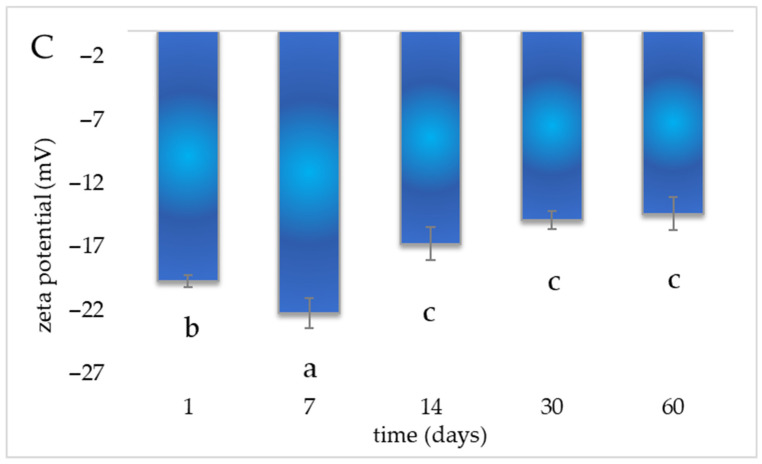
Storage stability of liposomal vesicles with encapsulated smoke tree extract and silibinin monitored for 60 days at 4 °C: (**A**) vesicle size, (**B**) polydispersity index (PDI), and (**C**) zeta potential; different letters show significant difference (*p* < 0.05; *n* = 3; analysis of variance, Duncan’s post hoc test).

The storage stability of liposomal vesicles with encapsulated STExt and SB was investigated over 60 days, as measured by physical properties of the developed liposomes, including particle size, size distribution, and zeta potential (Figure 7). The size and colloidal stability of the liposomes were evaluated by dynamic light scattering (DLS). DLS is a widely accepted technique for liposome characterization, as it provides a statistically representative average size of the entire liposome population in its native hydrated state [36]. In contrast, transmission electron microscopy (TEM) offers qualitative morphological information based on a limited number of dehydrated particles selected from representative images, which may not accurately reflect the overall size distribution in dispersion. Therefore, direct comparison between liposome sizes obtained by DLS and TEM is not straightforward, and DLS is particularly suitable for assessing liposome systems intended for biomedical applications [37,38]. The vesicle size of liposomes increased significantly during the first 30 days of storage (from 1978.7 nm to 4218.0 nm), followed by a slight decrease by the 60th day (3763.67 nm) (Figure 7A). These results are consistent with the formation of vesicular structures in aqueous dispersion. The observed size distribution and relatively low PDI values (~0.340) indicate a homogeneous vesicle population rather than random lipid aggregates. This increase can be attributed to the vesicle fusion or aggregation, a common phenomenon in liposomal systems during storage; instability of the lipid bilayer, potentially due to hydrolysis, oxidation, or phase transitions; and absorption of water into the bilayer or core, leading to swelling of the vesicles [39,40]. After reaching a peak size on the 30th day, the vesicles show a moderate size reduction by the 60th day (~10.8% decrease). This may be due to structural rearrangements of the lipid bilayer, possibly driven by thermodynamic stabilization over time; sedimentation or rupture of larger vesicles, which leaves behind smaller, more stable vesicles in the dispersion; and the release or leakage of encapsulated material, affecting vesicle integrity and size [41,42]. The changes suggest physical instability over time, particularly in the early phase of storage. While some maturation or stabilization may occur after 30 days, the system remains prone to significant size variation, which could influence the release behavior and bioavailability of the liposomal formulation. Therefore, the observed instability and changes in liposome size over time justify their incorporation into Pull-Iso films. Although the mean diameter of the liposomes used in the present study (~1979 nm) exceeds the size range typically considered optimal for transdermal penetration, the dermal delivery mechanism of this system does not rely on the penetration of intact liposomal vesicles through the *stratum corneum*. Instead, large multilamellar vesicles are expected to interact with the skin surface and act as reservoirs for the gradual release of the encapsulated bioactive compounds. Such non-penetrative mechanisms of dermal delivery by conventional liposomes have been widely reported in the literature [43,44]. Moreover, liposomal encapsulation serves primarily to protect sensitive bioactive compounds, such as SB and polyphenols, from degradation and to ensure their controlled availability when incorporated into the polymeric film matrix.

Additionally, in the present study, liposomes were intentionally used without size-reduction techniques to preserve their native multilamellar structure and stability immediately after preparation. Size reduction by probe sonication was avoided, as ultrasonic probes may introduce titanium residues into lipid vesicles, potentially affecting formulation purity and biocompatibility. This choice is supported by our previous in vitro investigations, in which the biological effects of multilamellar vesicles (MLVs) and unilamellar liposomes obtained by probe sonication (SUVs), both loaded with SB, were compared on skin cells [45]. In those experiments, unilamellar liposomes exhibited a pronounced cytotoxic effect on skin cells, whereas multilamellar vesicles did not show cytotoxicity. Importantly, both MLVs and SUVs demonstrated significant antioxidant and anti-inflammatory activity in vitro, suggesting that MLVs may represent a safer carrier system for dermal applications, while retaining the desired biological effects. This rationale supports the intentional use of relatively large multilamellar vesicles (~1979 nm) in the current study, as the dermal delivery mechanism relies on surface interaction and gradual release of bioactive compounds rather than penetration of intact vesicles through the *stratum corneum*. The PDI provides insight into the uniformity of particle size distribution, with values < 0.3 generally considered indicative of monodisperse systems, and values > 0.3 pointing to greater heterogeneity [42]. PDI values on the 1st day (0.340 ± 0.086) and the 14th day (0.360 ± 0.050) indicate a relatively broad size distribution, suggesting heterogeneity in vesicle formation or early-stage aggregation/fusion events (Figure 7B). The elevated standard deviations also reflect batch variability or instability in early formulation. A noticeable drop in PDI was observed on the 7th day (~0.250) and the 60th day (~0.260), both below the 0.3 threshold, indicating improved homogeneity in vesicle populations. The lowest PDI variability is seen at day 30th (0.340 ± 0.020), suggesting a temporary stabilization phase during mid-term storage. While the PDI fluctuates slightly over time, there is no sustained trend toward destabilization. These variations could be associated with minor fusion or breakdown events, reorganization of lipid bilayers, or sedimentation of larger vesicles, which can alter the population balance without drastically changing the average particle size [36]. Although the liposomal formulations showed moderate heterogeneity early on, the PDI values suggest improved colloidal stability after 30–60 days, likely due to lipid membrane rearrangement and distribution narrowing. Importantly, none of the values indicate severe aggregation or instability, supporting the overall structural integrity of the liposomes during the 60 days.

The zeta potential values of the samples were measured at multiple time points over 60 days, exhibiting an initial value of –19.7 ± 0.5 mV, followed by –22.23 ± 1.21 mV, –16.77 ± 1.31 mV, –14.87 ± 0.72 mV, and finally –14.40 ± 1.31 mV (Figure 7C). These results indicate a gradual reduction in the magnitude of the negative surface charge over time. Zeta potential is a key indicator of the electrostatic stability of colloidal systems, such as liposomal or nanoparticulate suspensions. Typically, values greater than ±30 mV are considered highly stable due to strong electrostatic repulsion preventing particle aggregation, while values between ±10 mV and ±30 mV suggest moderate stability, and values near zero imply potential instability and aggregation propensity [46]. In this case, the zeta potential values, although negative throughout, remain within the moderate stability range, suggesting that the samples maintain sufficient electrostatic repulsion to prevent significant aggregation during the 60-day storage period. However, the observed gradual decrease in negative charge magnitude (from approximately –22 mV to –14 mV) may indicate partial surface charge screening or changes in the surface chemistry of the particles. Possible causes include adsorption of ions or biomolecules from the storage medium, reorganization of surface groups, or slight particle aggregation [47]. The relatively low standard deviations suggest consistent measurement, reliability, and sample homogeneity at each time point. Despite the decline in zeta potential magnitude, the values do not approach neutrality, implying that the system retains colloidal stability and resistance to flocculation or sedimentation over the 60 days. The zeta potential trend reflects acceptable stability for the application considered, though the gradual charge reduction warrants monitoring, as prolonged storage or different environmental conditions may further impact surface charge and thus suspension stability. Namely, colloidal stability over time, as reflected by zeta potential measurements, additionally supports the presence of self-assembled lipid vesicles rather than non-specific aggregates. It should also be noted that microscopy and DLS measure fundamentally different aspects: microscopy visualizes a limited number of dried particles and may not capture the entire population, while DLS provides an intensity-weighted average of the hydrated population in solution. Therefore, while microscopy would offer complementary qualitative confirmation, the current combination of DLS-measured particle size, PDI, and zeta potential data provides strong and statistically representative evidence for the successful formation of liposomes.

Physicochemical characterization of liposomes after incorporation into the film matrix was not conducted using DLS measurements, as film redispersion would require mechanical forces that may compromise liposome integrity. Furthermore, the presence of film-forming components could interfere with the measurements, preventing reliable attribution of particle size, size distribution, and surface charge exclusively to liposomes.

### 3.5.Results of Optical Microscopy

**Figure 8:**
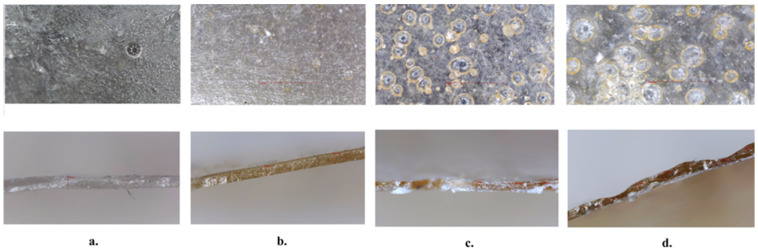
Optical micrographs of the surface and cross-section of the (**a**) neat Pull–Iso film, (**b**) Pull-Iso-Lip 1 film, (**c**) Pull-Iso-Lip 2 film, and (**d**) Pull-Iso-Lip 3 film.

Figure 8 presents the micrographs of neat Pull-Iso and liposome-loaded Pull-Iso films. Optical microscopy analysis revealed that optimal liposome incorporation was achieved in the Pull-Iso-Lip 2 sample, containing 25 wt% of liposome dispersion per biopolymer weight. A further increase in liposome content resulted in liposome aggregation and coalescence, which consequently deteriorated the mechanical properties of the films. Cohesive forces between liposomes predominated, preventing their uniform distribution within the matrix. The results of optical microscopy are consistent with the findings of the mechanical analysis (Section 3.8).

### 3.6. Results of Water Contact Angle Measurements

At initial contact with distilled water, the contact angle of the Pull-Iso film was 79.5°± 2.2, indicating a relatively hydrophobic surface. In contrast, the neat pullulan film exhibited a significantly lower contact angle of 39.2° ± 0.9, reflecting its pronounced hydrophilicity. This indicates that the esterification process imparted a more pronounced hydrophobic character to the polysaccharide, as reflected in the increased contact angle. This effect stems from the fact that the hydroxyl groups in the pullulan structure are replaced by hydrophobic alkenyl groups, which reduces the total surface energy of the material [48]. Considering the relatively high water contact angle of the skin surface (typically above 100°), the hydrophobic modification of pullulan may enhance the interfacial compatibility between the film and the skin, potentially providing favorable conditions for improved adhesion and performance in wound dressing applications [49]. The incorporation of liposomes into the Pull-Iso matrix led to a gradual decrease in surface hydrophobicity. Specifically, the contact angles measured for Pull-Iso-Lip 1 and Pull-Iso-Lip 2 films were 69.3° ± 2.2 and 63.1° ± 1.5, respectively, confirming the increased surface wettability induced by the liposomal components. However, at the highest liposome loading (Pull-Iso-Lip 3 film), a slight increase in the contact angle to 80.6° was observed, which could be attributed to liposome aggregation and the consequent exposure of hydrophobic domains on the film surface.

### 3.7. Results of Moisture Content (MC), Total Soluble Matter (TSM) Analysis, and Swelling Test

The results of MC, TSM, and SR are presented in Table 1. Esterification of pullulan decreases the number of hydroxyl groups available for hydrogen bonding with water [50], resulting in significantly lower MC and TSM values for the Pull-Iso film compared with neat pullulan. The SR could not be determined for the neat pullulan film, as it was completely dissolved in water.

The incorporation of liposomes up to an optimal concentration caused a slight decrease in SR and TSM, while MC increased moderately, which can be attributed to the hydrophobic lipid tails and encapsulated SB that alter the film’s water-binding and diffusion behavior. At higher liposome loadings, aggregation occurred, leading to the formation of microvoids that facilitated localized moisture absorption, thereby increasing both MC and SR. In contrast, the TSM increased progressively with higher liposome content, likely due to partial matrix disruption and the release of soluble components from aggregated liposomes. These findings are consistent with the observations from optical microscopy and mechanical testing, confirming the structural influence of liposome incorporation on film integrity and water interactions.

### 3.8. Mechanical Properties and Comparison with Literature

The mechanical properties of neat Pull–Iso and Pull–Iso films loaded with liposomes are presented in Table 2. Neat pullulan films typically exhibit high tensile strength and relatively low elongation at break under comparable test conditions, as reported in the literature for pullulan cast from aqueous solutions [50]. Chemical derivatization of pullulan significantly alters its mechanical behavior, as summarized in Table S1 (Supplementary Materials) [18,51,52,53]. Fully substituted pullulan acetate films produced by solvent casting retain comparatively high tensile strength (approximately 24 MPa), but show low elongation at break, indicating increased stiffness and reduced chain mobility. In contrast, pullulan fatty acid esters (hexanoate, octanoate, and decanoate), prepared via organic solvent casting without plasticizers, display markedly reduced tensile strength and low elongation at break [18]. This progressive deterioration in mechanical performance with increasing alkyl chain length is attributed to enhanced hydrophobicity, disrupted intermolecular hydrogen bonding, and poorer film cohesion.

Compared to parent pullulan films, Pull–Iso films display lower break stress, despite the same plasticizer content. This trend aligns with literature on polysaccharide fatty acid esters, where the introduction of long hydrophobic side chains generally weakens intermolecular cohesion in the polymer matrix [18]. Although the chemical nature and substitution pattern of Pull–Iso differ from simple alkyl esters, the trend of reduced tensile strength with increased hydrophobic modification is consistent. In our study, Pull–Iso films still retain tensile strength values substantially higher than those of many pullulan esters prepared via organic solvent casting, suggesting that the aqueous casting process and plasticizer choice can significantly affect the resultant mechanical performance. Specifically, the tensile strength of Pull–Iso films is up to ten times greater than that of certain pullulan hexanoate, octanoate, and decanoate films reported in the pullulan ester literature, which typically show low MPa-range strengths for long side-chain esters higher than for the neat Pull-based films. The incorporation of liposomes into Pull–Iso films results in a deterioration of mechanical properties, as evidenced by a reduction in both tensile strength and elongation at break. A further increase in liposome content results in a progressive decrease in these parameters. Overall, the influence of lipid compounds on the mechanical performance of films and coatings is highly dependent on both their chemical nature and concentration, as well as their compatibility with the polymer matrix. As reported by Zahedi et al. (2010) [51], the incorporation of fatty acids into pistachio globulin protein-based films led to a pronounced deterioration of mechanical performance, with elongation at break decreasing by 35–70% and a concomitant reduction in ultimate tensile strength. This behavior was attributed to the disruptive effect of low-molecular-weight fatty acids on protein–protein interactions, which promoted phase separation and weakened the film network. In contrast, Wang et al. (2016) [52] demonstrated that epoxy castor oil significantly enhanced the mechanical properties of soy protein-based films, with tensile strength nearly doubling and elongation at break increasing by approximately 23%. The improvement was ascribed to hydrogen bonding between the epoxide-containing long chains of epoxidized castor oil and soy protein isolates, which strengthened interfacial adhesion and improved stress transfer within the matrix.

### 3.9. Results of Antioxidant Capacity

**Figure 9:**
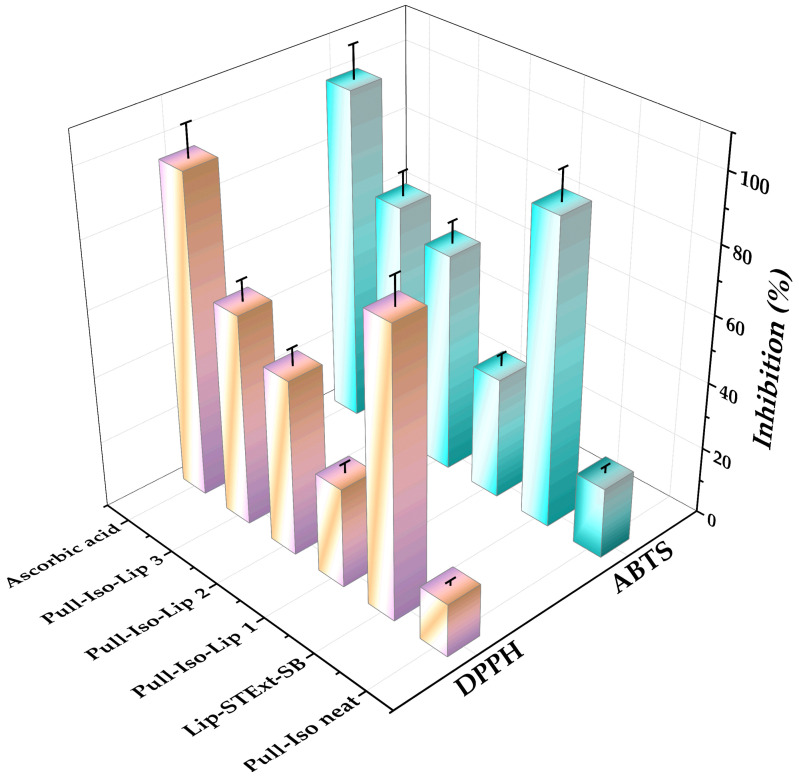
Antioxidant potential of neat Pull-Iso (pullulan-isononanoate), liposomes loaded with silibinin (SB) and smoke tree extract (STExt), and Pull-Iso films.

The combination of SB and STExt in the developed films is expected to produce a synergistic effect due to the complementary bioactivities of their constituent polyphenolic compounds. SB functions primarily as a potent antioxidant and free-radical scavenger, reducing oxidative stress in wounded tissue, as well as an anti-inflammatory and antimicrobial agent [53], while the complex mixture of polyphenols in STExt has been shown to exert additional antioxidant, anti-inflammatory, antimicrobial, and consequently tissue-repair-promoting effects [54]. The synergistic effect is hypothesized to arise from the combined antioxidant capacity and complementary modes of action of these compounds: while silibinin primarily quenches reactive oxygen species and stabilizes cellular membranes, the complex mixture of polyphenols in STExt may additionally modulate inflammatory mediators and enhance extracellular matrix remodeling. When co-delivered within the same film matrix, these compounds may provide a broader spectrum of protective and regenerative activity, leading to enhanced wound-healing performance compared to formulations containing either bioactive alone. This synergistic mechanism is supported by studies reporting enhanced bioactivity of combined polyphenolic systems in topical applications [54]. The antioxidant activity of the pullulan-based films was evaluated using the DPPH and ABTS radical scavenging assays, which are widely employed to assess hydrogen-donating and electron-transfer capacities, respectively (Figure 9). As expected, ascorbic acid, used as a reference antioxidant, exhibited the highest radical scavenging activity in both assays, confirming the validity and sensitivity of the applied methods. The control film without liposomes (Pull-Iso) showed relatively low antioxidant activity in both DPPH (~15.83%) and ABTS (~21.24%) assays. On the other hand, the liposomes with SB and STExt showed significant antioxidant potential toward DPPH (~84.9%) and ABTS (~91.3%) radicals. This limited activity can be attributed to the intrinsic antioxidant potential of the polymeric matrix itself, indicating that pullulan contributes only marginally to radical scavenging. However, the incorporation of liposomes loaded with STExt and SB significantly enhanced the antioxidant capacity of the films. A clear concentration-dependent increase in antioxidant activity was observed with increasing liposome content. Film Pull-Iso-Lip 1, containing 0.25 g of liposomes, demonstrated moderate radical scavenging activity (~29.78% for DPPH and ~36.55% for ABTS, Figure 9), indicating successful incorporation and partial availability of antioxidant compounds within the film matrix. Further enrichment with liposomes led to a pronounced improvement in antioxidant performance. Film Pull-Iso-Lip 2 (0.50 g liposomes) showed substantially higher activity, reaching ~52.47% and ~65.58% inhibition in DPPH and ABTS assays, respectively. The highest antioxidant capacity was recorded for film Pull-Iso-Lip 3, containing 0.75 g of liposomes, which exhibited ~63.03% DPPH and ~72.53% ABTS radical scavenging activity. This result confirms that higher liposome loading enables greater retention and release of bioactive compounds, such as phenolic constituents from STExt and SB, thereby enhancing the overall antioxidant potential of the biopolymer film. Plant polyphenols have attracted considerable scientific interest owing to their strong antioxidant properties, enabling them to function as reducing agents, hydrogen donors, and effective quenchers of singlet oxygen [6]. STExtis is rich in phenolic antioxidants [55], while SB is recognized for its strong antioxidant activity [56]; both contribute to increased antioxidant capacity in composite films. Namely, according to the previous study, HPLC-DAD analysis demonstrated that the ethanolic STExt was rich in flavonoid compounds, with fustin and sulfuretin being the most abundant, followed by butin, fisetin, taxifolin, and butein [57]. In all samples, ABTS values were consistently higher than those obtained with the DPPH assay. This difference may be attributed to the higher sensitivity of the ABTS assay toward both hydrophilic and lipophilic antioxidants, as well as its greater suitability for complex systems, such as liposome-loaded polymer films. The enhanced ABTS scavenging activity further suggests effective electron-transfer mechanisms mediated by encapsulated polyphenols [58]. The results demonstrate that the incorporation of liposomes encapsulating STExt and SB significantly improves the antioxidant properties of pullulan-based films in a dose-dependent manner. Such films may have potential for application as active packaging materials or biomedical films, where antioxidant activity could be beneficial for protection against oxidative degradation or oxidative stress. In all samples, ABTS values were consistently higher than those obtained with the DPPH assay. This difference may be attributed to the higher sensitivity of the ABTS assay toward both hydrophilic and lipophilic antioxidants, as well as its greater suitability for complex systems, such as liposome-loaded polymer films. The enhanced ABTS scavenging activity further suggests effective electron-transfer mechanisms mediated by encapsulated polyphenols [59]. The results demonstrate that the incorporation of liposomes encapsulating STExt and SB significantly improves the antioxidant properties of pullulan-based films in a dose-dependent manner.

## 4. Conclusions

Pullulan was successfully modified via direct esterification with isononanoic acid chloride, yielding branched Pull-Iso with enhanced structural flexibility. This chemical modification resulted in a notable reduction in the glass transition temperature, reflecting the increased free volume and segmental mobility of the polymer chains. Esterification also imparted pronounced hydrophobicity to the biopolymer, as evidenced by an increased water contact angle and reduced values of TSM and MC in a pH 5.5 buffer, corresponding to skin conditions. The incorporation of liposomes loaded with SB and STExt at an optimal concentration of 40 wt% relative to the biopolymer enhanced the film’s wettability, while maintaining a surface hydrophobicity comparable to that of human skin, ensuring favorable adhesion and comfort upon application. Microscopy and mechanical analyses confirmed that moderate liposome loading (Pull-Iso-lip 2) resulted in homogeneous morphology and optimal tensile properties. In contrast, higher concentrations led to liposome aggregation and a decrease in mechanical strength. The results confirm that liposome loading effectively enhances the antioxidant performance of Pull-Iso-based films by facilitating the retention and controlled release of polyphenolic compounds from STExt and SB. The consistently higher ABTS scavenging activity compared to DPPH further indicates efficient electron-transfer mechanisms in these complex, liposome-loaded film systems. These observations highlight the potential of such systems for active packaging and biomedical applications. Namely, these preliminary findings indicate that liposome-loaded Pull-Iso films may represent a promising platform for the development of flexible, bioactive-loaded wound dressings, warranting further biological validation to assess their potential. A limitation of the present study is the absence of compound-specific characterization of the plant extract and its encapsulation behavior, as advanced analytical techniques such as high-performance liquid chromatography (HPLC) were not applied. Future investigations will therefore focus on the detailed identification and quantification of individual extract constituents, together with the evaluation of their encapsulation efficiency within liposomal carriers and their distribution within the final film formulations. Nevertheless, the newly developed pullulan ester was comprehensively characterized in terms of its chemical structure, thermal stability, and film-forming performance in interaction with liposomes, thereby establishing a robust foundation for subsequent research. Future work will include in vitro assessment of these multifunctional films to evaluate their skin compatibility, bioactive release kinetics, and therapeutic efficacy in wound healing applications.

## Figures and Tables

**Table 1:** Moisture content (MC), total soluble matter (TSM), and swelling ratio (SR) values for neat Pull-Iso (pullulan-isononanoate) film and liposome-loaded Pull-Iso films.

Sample	MC (%)	TSM (%)	SR (%)
Pull	12.4 ± 1.2	95.4 ± 1.1	total soluble
Pull-Iso	7.5 ±1.4	58.4 ±1.9	62.1 ± 1.7
Pull-Iso-Lip 1	8.3 ± 1.7	55.7 ± 2.1	58.2 ± 2.2
Pull-Iso-Lip 2	10.2 ± 1.6	52.8 ± 1.8	45.8 ± 2.0
Pull-Iso-Lip 3	10.3 ± 1.8	51.9 ± 1.6	72.8 ± 1.4

**Table 2:** Break stress and break strain of neat Pull, Pull–Iso, and liposome-loaded Pull–Iso films.

Sample	Break Stress (MPa)	Break Strain (%)
Pull	22.75 ± 1.2	3.68 ± 1.1
Pull-Iso	20.97 ± 1.1	10.97 ± 0.9
Pull-Iso-Lip 1	10.85 ± 2.4	2.46 ± 2.1
Pull-Iso-Lip 2	8.79 ± 2.7	2.39 ± 2.5
Pull-Iso-Lip 3	5.26 ± 2.4	1.73 ± 2.7

## Data Availability

The original contributions presented in this study are included in the article/Supplementary Material. Further inquiries can be directed to the corresponding author.

## Supplementary Materials

The following supporting information can be downloaded at https://www.mdpi.com/article/10.3390/polym18020305/s1. Figure S1: 2D and 3D structure of pullulan; Figure S2. 2D and 3D structure of (isononanoic) 3,5,5-trimethylhexanoic acid; Figure S3. (a) ^1^H NMR, and (b) ^13^C NMR spectrum of pullulan; Figure S4. (a) FTIR, (b) ^1^H NMR, and (c) ^13^C NMR spectrum of 3,5,5-trimethylhexanoic acid; Figure S5. The structure of (a) phosphatidylcholine and (b) silibinin A. Figure S6. HSQC (top) and COSY (bottom) spectra of Pull-Iso (pullulan-isononanoate). Table S1. Comparison of mechanical properties of Pull–Iso films with literature data.

## Abbreviations

The following abbreviations are used in this manuscript:

STExt	Smoke tree extract
SB	Silibinin
TEMPO	2,2,6,6-tetramethyl piperidinyloxy
PHBHV	Poly(3-hydroxybutyrate-co-3-hydroxyvalerate
Pull-Iso	Pullulan isononanoate
ABTS	2,2′-azino-bis (3-ethylbenzothiazoline-6-sulphonic acid
DPPH	2,2-diphenyl-1-picrylhydrazyl
DMSO	Dimethyl sulfoxide
NMR	Nuclear magnetic resonance
FTIR	Fourier-transform infrared spectroscopy
DSC	Differential Scanning Calorimetry
AV	Acid value
SV	Saponification value
WCA	Water contact angle
MC	Moisture content
TSM	Total soluble matter
SD	Swelling degree
PDI	Polydispersity index
