# Supplementary material for: Design and Optimization of Pullulan-Isononanoate Films with Bioactive-Loaded Liposomes for Potential Biomedical Use
Source: Polymers (Basel). 2026 Jan 22;18(2):305. doi: 10.3390/polym18020305 (PMC12845936; doi:10.3390/polym18020305)
Supplement: Supplementary file 1 [file polymers-18-00305-s001.zip › polymers-4093606-supplementary.pdf]

Design and Optimization of Pullulan-Isononanoate Films with Bioactive-Loaded Liposomes for Potential Biomedical Use

Amjed A. Karkad [1,2], Aleksandar Marinković [1], Aleksandra Jovanović [3], Katarina Simić [4], Stefan Ivanović [4], Milena Milošević [4] and Tamara Erceg [5,*]

[1] Faculty of Technology and Metallurgy, University of Belgrade, Karnegijeva 4, 11120 Belgrade, Serbia; amjedkarkad85@gmail.com (A.A.K.); marinko@tmf.bg.ac.rs (A.M.)
[2] Faculty of Medical Technology, Elmergib University, Msallatah 7310500, Libya
[3] Institute for the Application of Nuclear Energy INEP, University of Belgrade, Banatska 31b, 11080 Belgrade, Serbia; ajovanovic@inep.co.rs
[4] Institute of Chemistry, Technology and Metallurgy—National Institute of the Republic of Serbia, University of Belgrade, Njegoševa 12, 11000 Belgrade, Serbia; katarina.simic@ihtm.bg.ac.rs (K.S.); stefan.ivanovic@ihtm.bg.ac.rs (S.I.); milena.milosevic@ihtm.bg.ac.rs (M.M.)
[5] Faculty of Technology Novi Sad, University of Novi Sad, Bulevar cara Lazara 1, 21000 Novi Sad, Serbia
* Correspondence: tamara.erceg@uns.ac.rs

3.1. Results of NMR analysis

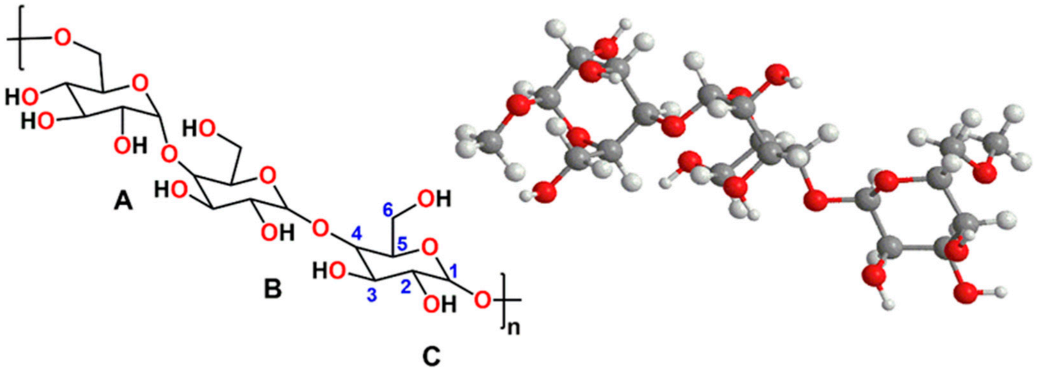

Figure S1. 2D and 3D structure of pullulan.

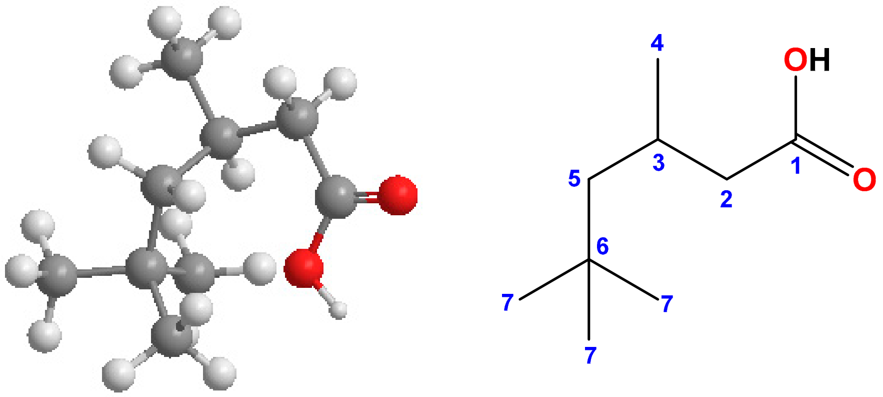

Figure S2. 2D and 3D structure of (isononanoic) 3,5,5-trimethylhexanoic acid.

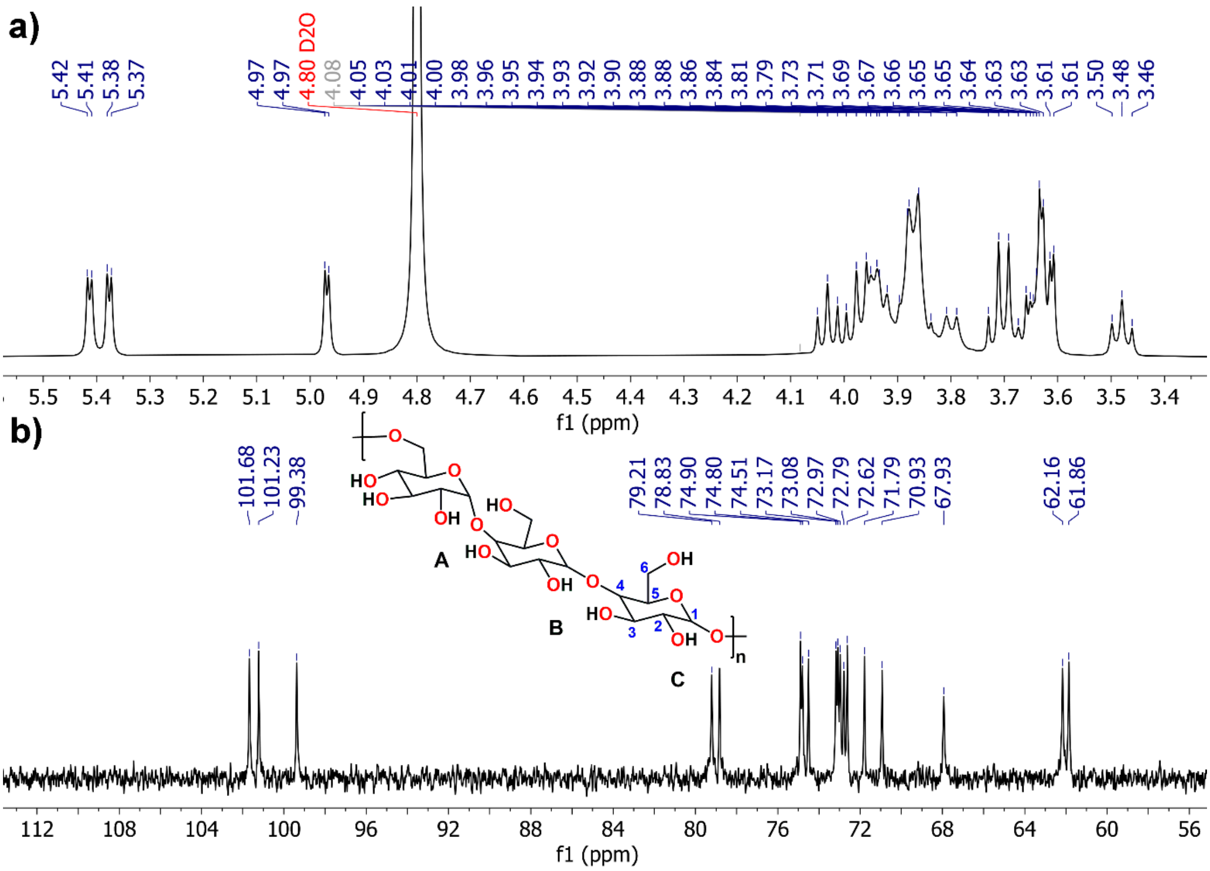

Figure S3. a) ^{1}H NMR, and b) ^{13}C NMR spectrum of pullulan.

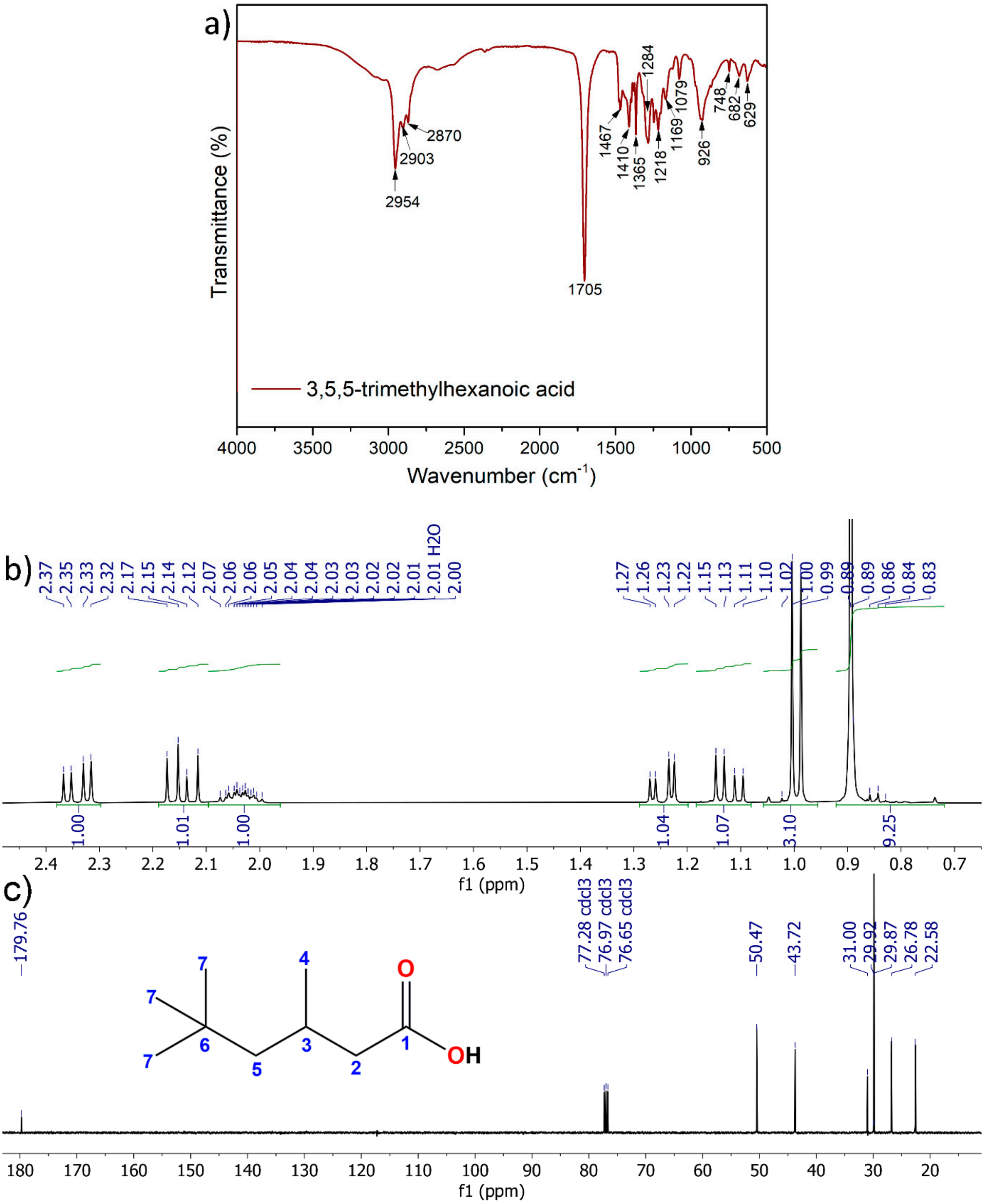

Figure S4. a) FTIR, b) ^{1}H NMR, and c) ^{13}C NMR spectrum of 3,5,5-trimethylhexanoic acid.

The results of FTIR, ^{1}H, and ^{13}C NMR indisputably confirm the success of the synthesis of 3,5,5-trimethylhexanoic acid. FTIR (ATR), cm^{-1}: 2954-2870 cm^{-1} ν(C–H stretching of –CH_3 and –CH_2 groups), 1705 cm^{-1} ν(C=O stretching), 1467-1365 cm^{-1} δ(C–H bending of –CH_3

and $-CH_2$ groups and O-H bending), 1161-926 cm^{-1} ν(C-O stretch). ^{1}H NMR ($CDCl_3$, 400 MHz, *δ*/ppm): 0.90 (9H, *s*, H-7), 1.00 (3H, *d*, *J*=6.6 Hz, H-4), 1.12 (1H, *dd*, J_1=14.0 and J_2=6.3 Hz, H-5) and 1.25 (1H, *dd*, J_1=14.0 and J_2=4.1 Hz, H-5), 2.04 (1H, *m*, H-3), 2.15 (1H, *dd*, J_1=14.8 and J_2=8.2 Hz, H-2) and 2.34 (1H, *dd*, J_1=14.8 and J_2=5.8 Hz, H-2). ^{13}C NMR ($CDCl_3$, 100 MHz, δ/ppm): 22.58 (C(4)), 26.78 (C(3)), 29.86 (3C(7)), 31.00 (C(6)), 43.72 (C(2)), 50.72(C(5)), 179.76 (C(1)).

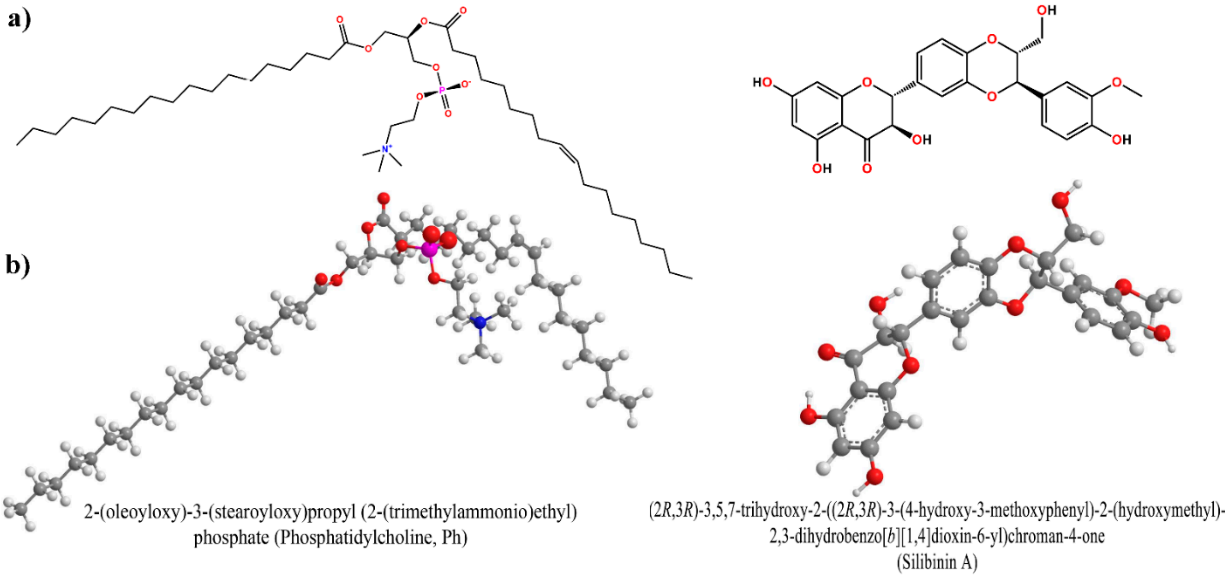

Figure S5. The structure of (a) phosphatidylcholine and (b) silibinin A.

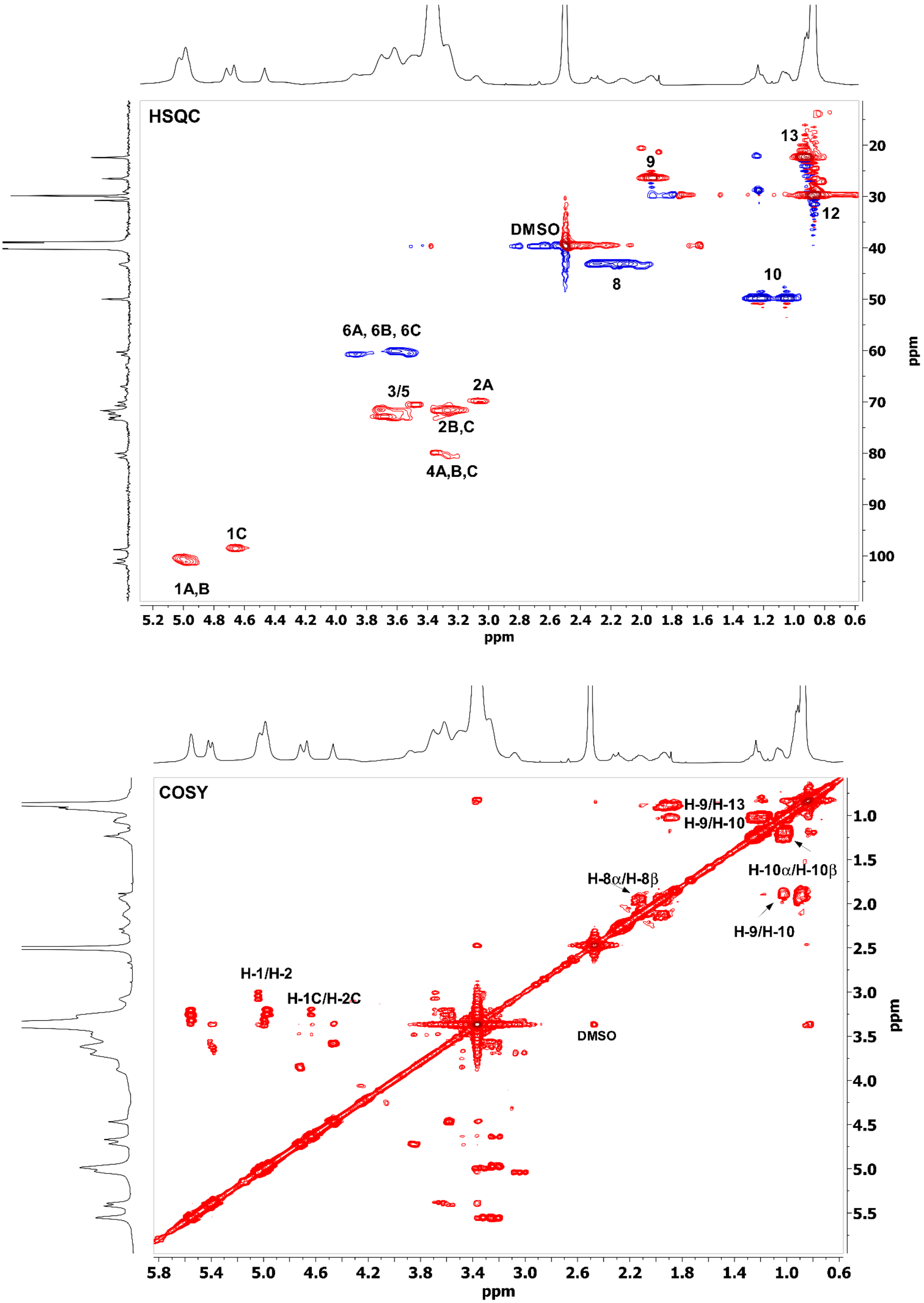

Figure S6. HSQC (top) and COSY (bottom) spectra of Pull-Iso (pullulan-isononanoate).

Table S1. Comparison of mechanical properties of Pull–Iso films with literature data

Material/system	Preparation method	Plasticizer/additive	Tensile strength/break stress	Elongation at break	Reference
Neat pullulan film	Aqueous casting	Glycerol	High (typically >20 MPa)	Low to moderate (≈3–5%)	IJBM, 2018, DOI: https://doi.org/10.1016/j.ijbiomac.2018.06.127
Pullulan acetate	Solvent casting	None/limited	High (≈20 MPa range)	Low	Eur. Polym. J., 2015, DOI: https://doi.org/10.1016/j.eurpolymj.2015.03.007
Pullulan hexanoate	Organic solvent casting	None	Low	Low	Eur. Polym. J., 2015, DOI: https://doi.org/10.1016/j.eurpolymj.2015.03.007
Pullulan octanoate	Organic solvent casting	None	Low	Low	Eur. Polym. J., 2015, DOI: https://doi.org/10.1016/j.eurpolymj.2015.03.007
Pullulan decanoate	Organic solvent casting	None	Very low	Low	Eur. Polym. J., 2015, DOI: https://doi.org/10.1016/j.eurpolymj.2015.03.007
Incorporation of fatty acids into pistachio globulin protein-based films	Solvent casting	None	Lower than pristine film	Lower than pristine film	Zahedi et al., 2010, 10.1016/j.jfoodeng.2010.03.033
Epoxy castor oil/soy protein-based films	Solvent casting	None	Improved	Significantly improved	Wang et al., 2026, 10.3390/ma9030193
